# Risk of second primary malignancies after definitive treatment for esophageal cancer: A competing risk analysis

**DOI:** 10.1002/cam4.2688

**Published:** 2019-11-15

**Authors:** Seiichiro Mitani, Shigenori Kadowaki, Isao Oze, Toshiki Masuishi, Yukiya Narita, Hideaki Bando, Sachiyo Oonishi, Yutaka Hirayama, Tsutomu Tanaka, Masahiro Tajika, Yutaro Koide, Takeshi Kodaira, Tetsuya Abe, Kei Muro

**Affiliations:** ^1^ Department of Clinical Oncology Aichi Cancer Center Hospital Nagoya Japan; ^2^ Department of Medical Oncology Kindai University Faculty of Medicine Osaka‐Sayama Japan; ^3^ Division of Cancer Epidemiology and Prevention Aichi Cancer Center Nagoya Japan; ^4^ Department of Endoscopy Aichi Cancer Center Hospital Nagoya Japan; ^5^ Department of Radiation Oncology Aichi Cancer Center Hospital Nagoya Japan; ^6^ Department of Gastroenterological Surgery Aichi Cancer Center Hospital Nagoya Japan

**Keywords:** competing risk analysis, esophageal cancer, second malignancies

## Abstract

**Background:**

Esophageal cancer is associated with synchronous or metachronous cancer at other primary sites. However, few studies have evaluated the second malignancies after the treatment of esophageal cancer. The present study aimed to clarify the frequency of and risk factors for the second malignancies after definitive therapy for esophageal cancer.

**Patients and Methods:**

We included patients with esophageal cancer who received definitive therapy between 2000 and 2010. Exclusion criteria were synchronous cancer or a past history of cancer. Standardized incidence rate (SIR) was calculated using age‐ and sex‐specific incidence rates from the cancer registry data. To conduct risk analyses, we used the competing risk regression model, which defined death and the development of second malignancies as competing risks.

**Results:**

A total of 758 patients were included, with 131 second malignancies occurring in 106 patients (14%), over a median follow‐up of 3.7 years. Cumulative incidences of second malignancies after 3, 5, and 8 years were 4.0%, 7.6%, and 13.8%, respectively. The risk of second malignancy was significantly elevated [SIR = 1.83, 95% confidence interval (CI): 1.50‐2.22]. The most common sites of primary tumor were the head and neck (20%), followed by the lung (17%), stomach (16%), colon and rectum (11%), and urinary tract (9%). Risk analyses revealed that age ≥ 65 years [subdistribution hazard ratio (sHR): 1.51, 95% CI: 1.01‐2.24, vs age < 65] and clinical stages 0‐I (sHR: 2.48, 95% CI: 1.46‐4.22, vs stage III and IV) and II (sHR: 2.10, 95% CI: 1.23‐3.58, vs stage III and IV) were significantly associated with second malignancies.

**Conclusions:**

Compared with the general population, an increased incidence of second malignancies was observed in the patients with esophageal cancer in the present study even after definitive treatment. Careful follow‐up is required, especially in patients at a higher risk of second malignancies.

## Graphical abstract

We conducted risk analyses using the competing risk regression model, which defined death and the development of second malignancies as competing risks. Our study revealed that risk of second primary malignancies was significantly higher compared with the general population. Age, clinical stage, and tumor location were identified as significant factors for the development of second primary malignancies.

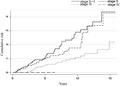

## 1 INTRODUCTION

Esophageal cancer is one of the most common cancers worldwide with approximately half a million new cases each year.^1^ Recent advances in multimodality treatment have prolonged survival outcomes of patients with esophageal cancer.^2^ Especially in the early stage of esophageal cancer, survival outcomes have recently improved as a result of the progress in early detection techniques and multimodality approach. In contrast to the Western countries, squamous cell carcinoma is the predominant histological type of esophageal cancer and accounts for approximately 90% of esophageal cancer cases in Japan.^3^, ^4^ The development of esophageal squamous cell carcinoma is closely associated with the alcohol assumption and smoking habits.^5^, ^6^ The loss of acetaldehyde dehydrogenase 2 (ALDH2), which is a key enzyme of alcohol metabolization, results in the accumulation of acetaldehyde and also increases the risk of esophageal squamous cell carcinoma.^7^ The concept of “field cancerization” proposes that multiple primary cancers simultaneously or metachronously occur with high rates, particularly in the head and neck region and in the esophagus.^8^, ^9^ In addition, alcohol consumption and smoking habits are common risk factors for several types of cancer. Previous studies have reported that 10% of patients with esophageal cancer have head and neck lesions and that screening for double cancer is recommended in pretreatment examination.^10^, ^11^ Conversely, a relatively fewer studies evaluating second primary malignancies (SPMs) after treatment of esophageal cancer have been reported. Two Japanese retrospective studies enrolled patients with esophageal cancer who underwent esophagectomy and suggested that SPMs were associated with high mortality.^12^, ^13^ Another study by Yamaguchi *et al* focused on patients with clinical stage II/III who received definitive chemoradiotherapy and achieved a complete response, thereby demonstrating that patients with esophageal cancer had a high incidence of SPM after chemoradiotherapy.^14^ A report from China analyzed patients with early stage esophageal squamous cell carcinoma who received esophagectomy and showed a distinctly high incidence of SPM.^15^ These retrospective studies raise an important issue of SPM for patients with esophageal cancer who were thought to be totally cured, but they included a relatively small number of patients, ranging from 93 to 679. Two pooled analyses of multiple cancer registries have shown an elevated risk of SPM after treatment for esophageal cancer.^16^, ^17^ Another population‐based study from Taiwan surveyed the largest cohort of 18,026 patients with esophageal cancer with a follow‐up of more than 15 years and demonstrated that the risk of metachronous SPM is significantly increased.^18^ However, these studies lacked detailed patient characteristics such as alcohol consumption or smoking habits, which can affect cancerogenesis despite the large number of patients studied. Additionally, all these studies did not consider the survival outcomes of esophageal cancer. It is natural that a poor prognosis in primary cancer leads to a short follow‐up, which is less likely to cause a second malignancy. In the case of evaluating SPMs, standard analysis methods such as the Kaplan‐Meier method or Cox proportional hazard models are designed to target only one type of event—the development of SPMs. However, in the real‐world setting, subjects can potentially develop the other types of events. In this case, mortality due to esophageal cancer or other diseases can affect the incidence of SPM, which is referred to a competing risk. Thus, a special type of method using a competing risk analysis is essential for appropriate evaluation of SPM. In this context, the present study aimed to clarify the frequency and risk factors for SPMs after definitive treatment for esophageal cancer.

## 2 MATERIALS AND METHODS

### 2.1 Identification of patients

We retrospectively reviewed a computerized database of patients with esophageal cancer treated at Aichi Cancer Center Hospital between January 2000 and December 2010. We included patients with histologically confirmed esophageal cancer who received definitive therapies with curative intent and excluded the patients with metastatic lesions who underwent palliative therapy, with synchronous cancer from other organs, or with a past history of cancer. This study was approved by the Institutional Review Board of Aichi Cancer Center Hospital, and all the patients provided informed consent for this study.

### 2.2 Statistical analyses

Patient demographic characteristics were based on medical records at the time of initial diagnosis. Treatment was defined as an initial‐phase therapy used for esophageal cancer and divided into the following four modalities: chemotherapy, surgery, radiotherapy, and endoscopic therapy. If patients underwent more than two modalities, these were counted as overlapped. Primary tumor locations in the esophagus were divided into three parts. The upper third included the cervical esophagus and thoracic upper esophagus, the lower third included the thoracic lower esophagus and abdominal esophagus, and the thoracic middle esophagus was classified as the middle third. Clinical stage was determined according to Unio Internationalis Contra Cancrum (UICC) 7th edition.^19^ History of smoking or alcohol was retrospectively evaluated from the records of the routine questionnaire. Social drinkers were considered as nondrinkers. SPMs were defined as a newly diagnosed cancer (other than esophageal cancer) after treatment initiation for esophageal cancer. Primary malignancies that developed within 6 months following the diagnosis of esophageal cancer were excluded from SPMs. Squamous cell carcinoma developing in the lung were also excluded because it is extremely difficult to discriminate lung metastases from esophageal cancer and primary lung carcinoma. Follow‐up period was defined as the number of years from the diagnosis of esophageal cancer to either the date of death from any cause, the date of diagnosis of SPM, or date of last follow‐up, whichever came first. When we consider the risk of SPM, death is a competing event. Unlike censoring, competing event could not occur together with the event of interest. Therefore, unbiased hazard ratios could not be calculated by conventional survival analysis. Competing risk regression is a useful alternative to Cox regression when considering competing events. Risk of SPM was assessed by subdistribution hazard ratio calculated by competing risk regression model, which defined a competing event as death from any cause and development of second malignancies.^20^, ^21^ The risk of SPM in patients with esophageal cancer was estimated using standardized incidence rate (SIR), which is a ratio of an observed to an expected number of patients with SPMs. The expected number of SPM during the person‐years at risk was determined on the basis of sex, age, and calendar‐year specific incidence rates from the Aichi Cancer Registry data from 2000 and 2014.^22^ Person‐years at risk was calculated using calendar‐year and each patient's follow‐up period. Cumulative incidence was calculated using the Kaplan‐Meier method. STATA version 15 (STATA Corp.) was used for all statistical analyses. All tests were two‐sided, and *P* < .05 was considered statistically significant.

## 3 RESULTS

### 3.1 Patient characteristics

**Figure 1:**
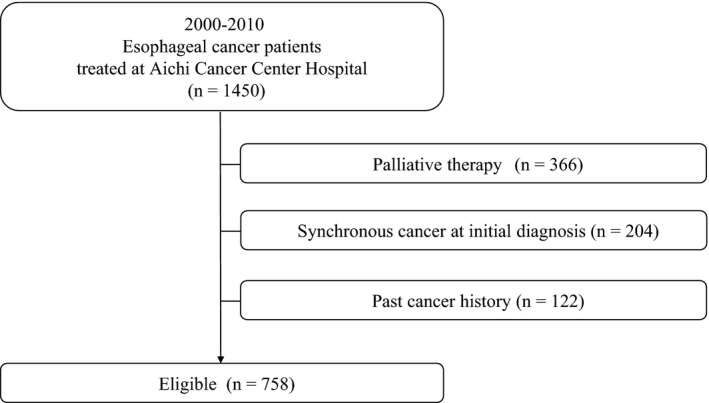
Study flow chart

We identified 1450 patients with esophageal cancer who were treated at Aichi Cancer Center Hospital. We excluded 366 patients who received nondefinitive treatment or palliative therapy such as chemotherapy in the metastatic setting. Moreover, 204 patients with synchronous cancer at initial diagnosis and 122 patients with a past history of cancer were excluded. Finally, the total study cohort comprised 758 patients (Figure 1).

Table 1 summarizes the characteristics of patients with esophageal cancer as a first primary or second primary cancer. Treatment modalities used as definitive treatment are listed in Table 2. Chemotherapy, surgery, radiotherapy, and endoscopic therapy were performed in 579 (76%), 374 (49%), 349 (46%), and 107 (14%) patients, respectively. A total of 583 patients (77%) received treatment using more than two modalities.

**Table 1:** Patient characteristics

	Patients (n = 758)
Age, years	
Median (range)	64 (32‐84)
Sex	
Male	634 (84%)
Female	124 (16%)
Primary tumor location in the esophagus	
Upper third	151 (20%)
Middle third	372 (49%)
Lower third	235 (31%)
Histology	
Squamous cell carcinoma	715 (94%)
Adenocarcinoma	30 (4%)
Others	13 (2%)
Clinical stage (UICC 7th)	
0–I	213 (28%)
II	183 (24%)
III	343 (45%)
IV	19 (3%)
History of alcohol	
Yes	657 (87%)
No	101 (13%)
History of smoking	
Yes	650 (86%)
No	108 (14%)
Treatment modality as initial therapy	
Chemotherapy	579 (76%)
Surgery	374 (49%)
Radiotherapy	349 (46%)
Endoscopic therapy	107 (14%)

Abbreviation: UICC, Unio Internationalis Contra Cancrum.

**Table 2:** Number of patients and SIRs for main malignancies according to sites

Site of SPM	n	SIR (95% CI)
All organs	132	1.83 (1.50‐2.22)
Head and neck	27	11.92 (7.58‐18.57)
Lung	22	1.67 (0.99‐2.77)
Gastric	21	1.94 (1.20‐3.10)
Colorectal	15	1.34 (0.76‐2.31)
Urinary tract	13	1.84 (0.86‐3.79)

Abbreviation: SPM, second primary malignancy.

### 3.2 Cumulative incidence of second primary malignancies

**Figure 2:**
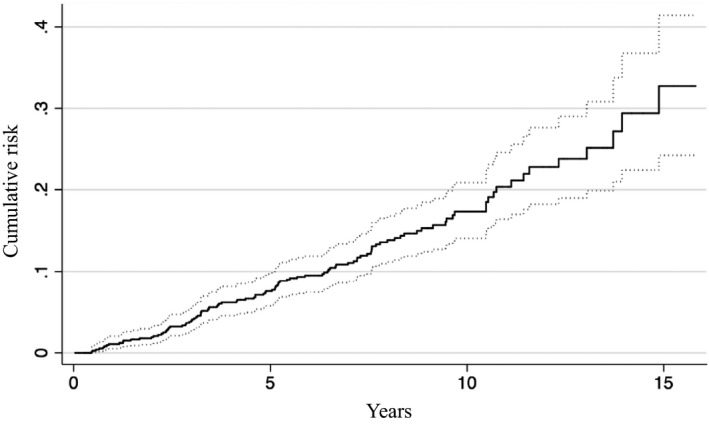
Cumulative incidence of second malignancies

A total of 132 SPMs occurred in 107 patients (14%) with a median follow‐up of 3.7 years (range 0.1‐16.8 years). Cumulative incidences of SPMs from all organs after 3, 5, and 8 years were 4.0%, 7.6%, and 13.8%, respectively (Figure 2). Compared with the general population in Aichi Prefecture, patients with esophageal cancer who received definitive treatment had a significantly increased risk of overall SPMs [SIR 1.83, 95% confidence interval (CI) 1.50‐2.22]. The most common primary tumor sites were the head and neck (n = 27), followed by the lung (n = 22), stomach (n = 21), colon and rectum (n = 15), urinary tract (n = 13), prostate (n = 9), and leukemia (n = 7). Of note, SIRs were significantly higher for head and neck cancer (11.92, 95% CI 7.58‐18.57) than for gastric cancer (1.94, 95% CI 1.20‐3.10). A nonsignificant trend toward higher SIRs was observed for lung (1.67, 95% CI 0.99‐2.77), colorectal (1.34, 95% CI 0.76‐2.31), and urinary tract cancers (1.84, 95% CI 0.86‐3.79). In total, 369 patients died, and the most common cause of death was related to primary esophageal cancer (n = 305). A total of 32 patients (4%) died due to SPMs, which was the second most common cause of death.

### 3.3 Risk analyses with competing risk regression model

**Figure 3:**
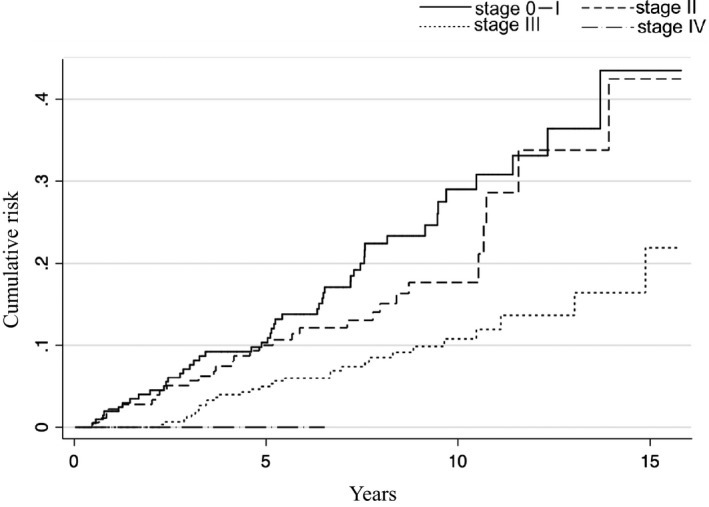
Cumulative incidence of second malignancies according to clinical stages

Results of SPM risk analyses using the competing risk regression model are presented in Table 3. Older age (age ≥ 65) was associated with the development of SPMs [subdistribution hazard ratio (sHR): 1.51, 95% CI: 1.01‐2.24, *P* = .04]. In addition, clinical stages 0‐I (sHR: 2.48, 95% CI: 1.46‐4.22, *P* = .001) and II (sHR: 2.10, 95% CI: 1.23‐3.58, *P* = .007) were associated with a higher incidence of SPM compared with clinical stages III and IV. Regarding primary tumor location in the esophagus, significant results were obtained for the middle third compared with the upper third (sHR: 1.94, 95% CI: 1.02‐3.70, *P* = .04). However, no significant association was observed between SPMs and treatment modalities. The 5‐year cumulative incidence of SPM was 10% in clinical stage 0–I, 10% in clinical stage II, and 5% in clinical stage III. The 8‐year cumulative incidence of SPM was 22% in clinical stage 0–I, 15% in clinical stage II, and 9% in clinical stage III (Figure 3).

**Table 3:** Risk analyses using the competing risk regression model

Factors	SHR	95% CI	*P*
Age, years (vs < 64 y)			
≥65	1.51	1.01‐2.24	.04
Sex (vs Male)			
Female	0.73	0.32‐1.66	.45
Histology (vs Nonsquamous)			
Squamous	1.43	0.54‐3.78	.47
Primary tumor location (vs Upper)			
Middle	1.94	1.02‐3.70	.04
Lower	1.79	0.89‐3.57	.10
Treatment (vs None)			
CT or RT	0.64	0.36‐1.14	.13
Both CT and RT	1.06	0.65‐1.72	.82
Clinical stage (vs stage III‐IV)			
0–I	2.48	1.46‐4.22	.001
II	2.10	1.23‐3.58	.007
History of smoking (vs No)			
Yes	0.57	0.17‐1.93	.37
History of alcohol (vs No)			
Yes	0.55	0.16‐1.87	.34

Abbreviations: CT, chemotherapy; RT, radiotherapy; SHR, subdistribution hazard ratio

## 4 DISCUSSION

In this study, we demonstrated that patients with esophageal cancer are still at a high risk of SPM even after definitive treatment. We determined that age, clinical stage, and tumor location were associated with the development of SPMs. To the best of our knowledge, this is the first study to identify significant risk factors for SPMs using a competing risk regression model. A competing risk analysis is a special type of survival analysis method that estimates the probability of an event in the presence of competing events.^20^, ^21^ For the aforementioned reasons, competing risk analyses were essential to evaluate SPMs in the presence of poor survival of esophageal cancer. Moreover, to the best of our knowledge, the present study had the largest study cohort among reported studies, except for pooled analyses of cancer registry and population‐based studies.

The risk of overall SPMs was significantly higher in the patients in the present study than in the general population (SIR 1.83, 95% CI 1.50‐2.22), which is comparable with the results reported in previous studies. Hu, et al and Chen, et al have reported higher SIRs of 3.84 (95% CI 2.98‐4.95) and 3.53 (95% CI 3.30‐3.77), respectively. However, in the present study, individual patient data were reviewed in more detail compared with previous pooled analyses or population‐based studies. In addition, patient background and, in particular, regional differences, may account for these discrepancies. Despite varying SIRs, SPMs have consistently been shown to be a main problem even after treatment for esophageal cancer.

Consistent with previous reports, the most common primary tumor sites of SPMs were the aerodigestive tract organs, such as the head and neck, lung, and stomach. In particular, the SIR of head and neck cancer was extremely high possibly because of the “field cancerization” concept, which claims carcinogenic effects of tobacco and alcohol on the aerodigestive tract simultaneously and an increased risk of the development in involved organs.^8^ Lung cancer was the second leading SPM despite exclusion of squamous cell carcinomas in the lung. Special attention for these types of cancer is definitely needed in addition to the detection of recurrence of esophageal cancer when follow‐up examinations, such as CT or upper endoscopy, are performed. Conversely, a high incidence of cancer in the nonaerodigestive tract, such as the colorectal and urinary tract cancers, was also observed, although not significant. Since these types of cancers are not routinely surveyed during follow‐up for esophageal cancer, extra screening examinations are required for their early diagnosis. The optimal surveillance for SPMs over long term should be clarified in future research.

In the present study, we identified age, clinical stage, and tumor location as significant factors for the development of SPM. Notably, compared with clinical stages III and IV, early stage (stage 0‐I sHR: 2.48, stage II sHR: 2.10) was associated with a higher incidence of SPM. It is reasonable that patients with early stage cancer have good prognosis and, therefore, are at a greater risk of developing SPM over a longer survival period. The reason for increased risk of SPMs in the middle third esophagus can be explained by the association similar to that between esophageal cancer in upper third esophagus and worse prognosis as reported previously.^23^ These findings indicated the importance of SPMs, especially in patients with esophageal cancer with good prognosis. Meanwhile, previous reports have shown that younger age, male sex, family history of cancer, liver cirrhosis, prior radiotherapy, prior surgery, prior chemotherapy, alcohol consumption, and smoking habits are significant risk factors for SPMs, but early stage has not previously been proposed as a risk factor.^12^, ^13^, ^15^, ^16^, ^17^, ^18^ This is probably because previous reports have not analyzed patient data using competing risk analysis. In standard analyses using Cox model, all events are assumed to be independent and death from any cause is censored. However, the development of SPM and death are not independent in clinical setting. A strength of the present study is the identification of SPM risk factors using a competing risk analysis, which is truly important in clinical settings. With regard to age, a population‐based study by Chen et al have proposed that younger age (<60 years) was correlated with SPMs. In general, older age is associated with a high risk of developing any type of cancer.^24^ As Chen et al did not analyze detailed patient characteristics, some confounding factors might result in a higher risk among a younger population.

There are several limitations in this present study that warrant discussion. First, this was a retrospective analysis from a single institution. Moreover, the study included a sample size of 758 patients and median follow‐up of 3.7 years, which may be insufficient to evaluate SPMs. The findings of the present study should be validated further in future research. Second, we defined treatment modality as an initial‐phase therapy used for esophageal cancer, and thus, treatment for relapse after initial therapies was not included in the risk analyses. However, we believe that the prognosis of esophageal cancer rather than SPMs has importance in patients with recurrence. Third, almost all patients with squamous cell carcinoma had a past history of alcohol and smoking. In addition, it is unclear whether these patients continued alcohol consumption or tobacco smoking, and thus, risk analyses of such indicators were not performed. However, despite these limitations, we believe that the present study is the first to report significant risk factors for SPMs after definitive therapies and demonstrate that early stage is highly associated with the development of SPMs using a competing risk analysis.

In summary, patients with esophageal cancer still have a high risk of second malignancies after completing primary treatment. Early stage and primary tumor in the lower portion of the esophagus were significant risk factors for SPMs on the basis of the competing risk analysis. This is possibly because patients with good prognosis have a greater risk of developing SPMs over a longer survival period, indicating an association between good prognosis of esophageal cancer and the development of SPMs. Older age was also identified as a risk factor for SPMs. Careful follow‐up is particularly required in such patients. Prospective trials to determine the optimal surveillance strategies are urgently required. We are planning to conduct a further study of SPMs using data from JCOG0502 (UMIN000000551) with an aim of comparing esophagectomy with definitive chemoradiotherapy for T1bN0 esophageal cancer. In that trial, all recruited patients had esophageal cancer with early stage and received endoscopic examination and CT scan at a designated interval during post‐treatment follow‐up period in this prospective study. We can gain new insights regarding surveillance strategies.

## AUTHOR CONTRIBUTION

Seiichiro Mitani, Shigenori Kadowaki, and Isao Oze conceptualized and designed the study. Seiichiro Mitani, Sachiyo Onishi, Yutaka Hirayama, Tsutomu Tanaka, Masahiro Tajika, Yutaro Koide, Takeshi Kodaira, Tetsuya Abe, and Kei Muro involved in acquisition of data. Seiichiro Mitani, Isao Oze, Shigenori Kadowaki, Toshiki Masuishi, Yukiya Narita, Hideaki Bando, and Kei Muro involved in analysis and interpretation of data. All the authors were involved in manuscript writing and gave final approval and agreed to be accountable for all aspects of the work.
